# Gabapentin Administration Reduces Reactive Gliosis and Neurodegeneration after Pilocarpine-Induced Status Epilepticus

**DOI:** 10.1371/journal.pone.0078516

**Published:** 2013-11-08

**Authors:** Alicia Raquel Rossi, Maria Florencia Angelo, Alejandro Villarreal, Jerónimo Lukin, Alberto Javier Ramos

**Affiliations:** Instituto de Biología Celular y Neurociencia “Prof. E. De Robertis”, Facultad de Medicina, Universidad de Buenos Aires, Ciudad de Buenos Aires, Argentina; Universidade de São Paulo, Brazil

## Abstract

The lithium-pilocarpine model of epilepsy reproduces in rodents several features of human temporal lobe epilepsy, by inducing an acute status epilepticus (SE) followed by a latency period. It has been proposed that the neuronal network reorganization that occurs during latency determines the subsequent appearance of spontaneous recurrent seizures. The aim of this study was to evaluate neuronal and glial responses during the latency period that follows SE. Given the potential role of astrocytes in the post-SE network reorganization, through the secretion of synaptogenic molecules such as thrombospondins, we also studied the effect of treatment with the α2δ1 thrombospondin receptor antagonist gabapentin. Adult male Wistar rats received 3 mEq/kg LiCl, and 20 h later 30 mg/kg pilocarpine. Once SE was achieved, seizures were stopped with 20 mg/kg diazepam. Animals then received 400 mg/kg/day gabapentin or saline for either 4 or 14 days. In vitro experiments were performed in dissociated mixed hippocampal cell culture exposed to glutamate, and subsequently treated with gabapentin or vehicle. During the latency period, the hippocampus and pyriform cortex of SE-animals presented a profuse reactive astrogliosis, with increased GFAP and nestin expression. Gliosis intensity was dependent on the Racine stage attained by the animals and peaked 15 days after SE. Microglia was also reactive after SE, and followed the same pattern. Neuronal degeneration was present in SE-animals, and also depended on the Racine stage and the SE duration. Polysialic-acid NCAM (PSA-NCAM) expression was increased in hippocampal CA-1 and dentate gyrus of SE-animals. Gabapentin treatment was able to reduce reactive gliosis, decrease neuronal loss and normalize PSA-NCAM staining in hippocampal CA-1. In vitro, gabapentin treatment partially prevented the dendritic loss and reactive gliosis caused by glutamate excitotoxicity. Our results show that gabapentin treatment during the latency period after SE protects neurons and normalizes PSA-NCAM probably by direct interaction with neurons and glia.

## Introduction

Epilepsy, a disorder characterized by recurrent seizures, is the second most common neurological disorder, affecting more than 50 million people worldwide [1]. Animal models of epilepsy and studies on human tissue from epileptic patients have shown that epileptogenesis involves a number of cellular and molecular events including alterations in brain circuitry, changes in gene expression, neurochemical alterations in receptors and neurotransmitters, aberrant neurogenesis and extended neuronal death [2]; [3]. Currently available pharmacological treatments are not disease-modifying and only suppress seizures, therefore being anti-epileptic but not anti-epileptogenic [4].

Recent experimental and clinical evidence has shown that the activation of innate immunity and inflammatory pathways contributes to the development of epilepsy [5]; [6]. It is also known that microglial and astroglial cells are the main effectors of the innate immune system in the brain. Activation of innate immunity can be achieved by molecular patterns derived from pathogens (Pathogen Associated Molecular Patters, PAMPs), and also by the release of endogenous intracellular molecules induced by tissue damage or cellular stress. Molecules of the latter kind are collectively known as Damage Associated Molecular Pattern (DAMP), and they efficiently activate pattern recognition receptors (PRR) of different families, including toll-like (TLR) and the Receptor for Advanced Glycation End products (RAGE).

Astrocytes play a major role in the regulation of the inflammatory response in different human Central Nervous System diseases, including epilepsy. Astrocytes and microglia respond to neuronal alterations and brain damage with a generic response of reactive gliosis, that involves secretion of proinflammatory cytokines and chemokines [7]. Several lines of evidence show that epilepsy is related to inflammation and increased levels of cytokines. In fact, virtually all the proinflammatory cytokines are elevated in the cerebrospinal fluid and brain tissue of epileptic patients (reviewed in [8]). A number of studies performed on epileptic subjects have shown that cytokine level correlates with the intensity and duration of epileptic seizures [9], [10]. Reactive gliosis is also associated with secondary epilepsy after traumatic or ischemic brain injury, as well as with major epilepsy related pathologies such as hippocampal sclerosis and focal malformations of cortical development [11]. Experimental models have provided additional evidences of the astroglial participation in epileptogenesis. Reactive gliosis was a common finding after different experimental paradigms of epilepsy [12]; [13]; [14]. However, the loss of astrocytes after severe epileptic seizures was also found [15]; [16]; [17], a fact that suggest that astrocytic malfunctioning could also induce secondary neuronal loss [18]. Microglia and brain resident dendritic cells also become activated in experimental models of epilepsy [19].

Regarding the study of the specific signaling pathways involved, the activation of NF-κB transcription factor regulates the expression of several molecules that control inflammation and cell survival. Induction of NF-κB-dependent transcription is a common response after the activation of IL1R1/TLR pathways, and has a major role in the control of innate immunity responses, as it governs the expression of genes encoding downstream mediators of inflammation, including IL-6, TNF-α, cyclooxygenase-2 (Cox-2), or CCL2 (i.e., monocyte chemotactic protein-1) (reviewed in [11]). Evidence from epileptic human tissue has pointed out to the probable activation of the NF-κB pathway in glia [20]; [21].

As regards pharmacological strategies, gabapentin was originally developed as an anticonvulsant drug that may act as a GABA-analog, but recent studies have shown that it is able to block the interaction of glial-derived thrombospondin 1–2 (TSP1–2) with the α2δ1 receptors that regulate excitatory synapses formation [22]; [23]. On the other hand, gabapentin was demonstrated to be an efficient drug for the control of neuropathic pain [24]. It has been recently shown that its effect may be related to the expression of α2δ1 receptors in the glial cell population and suppression of microglial reactive response [24].

In experimental models of TLE, the silent period between the primary injury induced by SE and the onset of spontaneous epileptic seizures, usually 21–30 days in the lithium pilocarpine model of epilepsy, comprises several molecular and cellular processes that are still obscure. Using the lithium-pilocarpine model, our aim was to study this early period of the epileptogenesis and to assess whether gabapentin is able to disrupt the early astroglial and microglial response to SE.

## Materials and Methods

### Ethics Statement

All procedures involving animals and their care were conducted in accordance with our institutional guidelines, which comply with the NIH guidelines for the Care and Use of Laboratory Animals and the principles presented in the Guidelines for the Use of Animals in Neuroscience Research by the Society for Neuroscience, and were approved by the CICUAL committee of the School of Medicine, University of Buenos Aires (Res. Nr. 1278/2012). All efforts were made to minimize animal suffering and to reduce the number of animal used.

### Materials

Primary Antibodies were purchased from Dako (anti-Glial Fibrillary Acidic Protein, GFAP); Millipore-Chemicon (anti-NeuN, anti-PSA-NCAM, anti-MAP2); Santa Cruz (anti-NF-κB p65NLS) and Hybridoma Bank of the Iowa University (anti-Nestin). Secondary fluorescent antibodies were from Jackson Immunoresearch. Tomato-Lectin FITC, Fluoro Jade B, biotinilated secondary antibodies, extravidin complex, glutamate and other chemicals were obtained from Sigma (St. Louis, MO). Gabapentin (Neurontin) was from Pfizer (Argentina). DMEM, antibiotics and glutamine were purchased from Invitrogen (Carlsbad, CA); fetal calf serum was obtained from Natocor (Cordoba, Argentina).

### Animal Procedures

**Figure 1:**
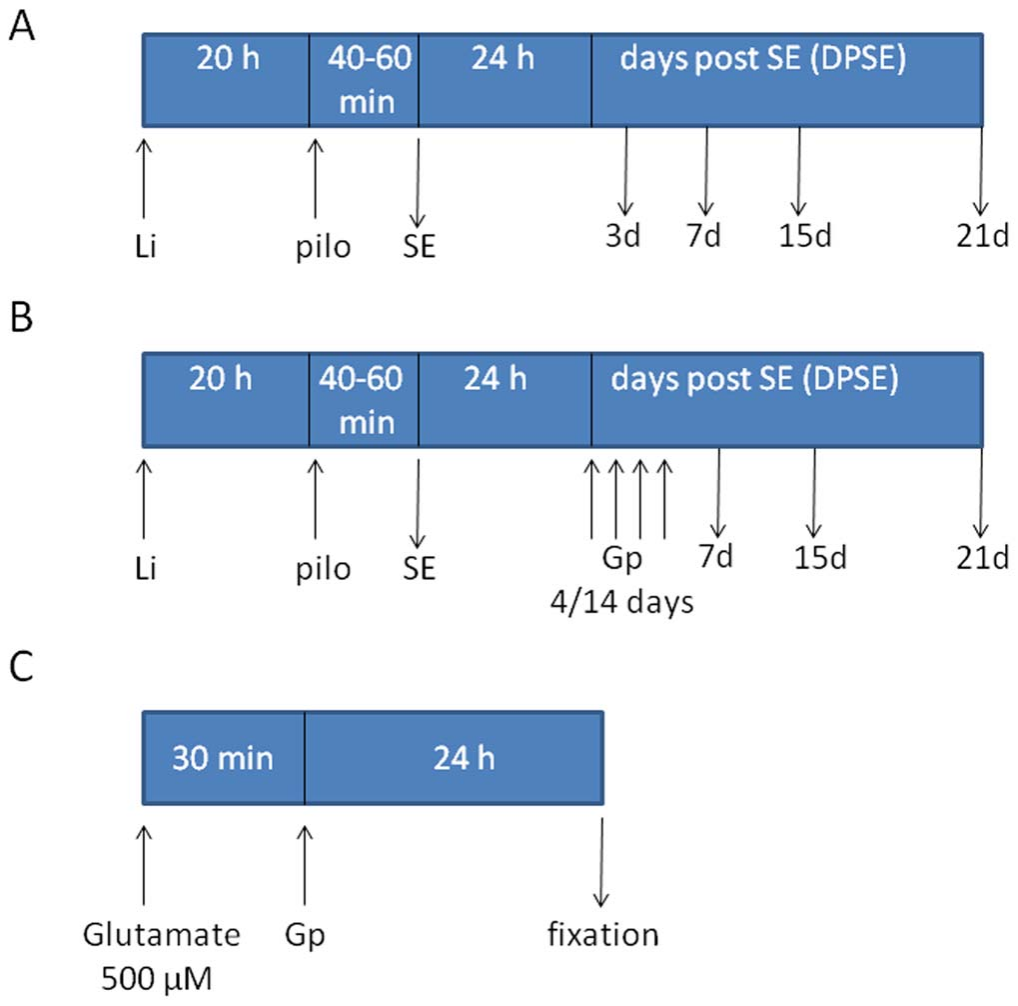
Schematic representation of the in A: In vivo lithium-pilocarpine exposure to describe neuro-glial alterations induced by the experimental model. B: Gabapentin treatment preformed on animals exposed to the lithium-pilocarpine experimental model. C: In vitro paradigm to study gabapentin effects on dissociated mixed cell culture from postnatal rat hippocampi. Li: Lithium chloride; pilo: pilocarpine; Gp: gabapentin; SE: status epilepticus. See material and methods for details.

Adult male Wistar rats (200–250 gr) from the animal breeding facility of the School of Pharmacy and Biochemistry (University of Buenos Aires) were used in this study. Animals were housed under controlled temperature, humidity and lighting conditions (20–25°C, 60% humidity, 12 h/12 h light/dark cycle) with standard laboratory rat food and water *ad libitum,* under the permanent supervision of a professional technician. Rats were subjected to the lithium-pilocarpine model of Temporal Lobe Epilepsy (TLE) (reviewed in [25]). Animals were randomly divided into three experimental groups. Two groups were injected with 3 mEq/kg lithium chloride (LiCl) intraperitoneally (i.p.); the control group received an equivalent volume of saline. Twenty hours later animals received either saline i.p. or 30 mg/kg pilocarpine (Li-pilo group). Animals that received saline-saline (control group) or Li-saline did not show significant differences either in behavioral or morphometric parameters, so to simplify images and graphs, only Li-saline controls are shown. Behavioral seizures were evaluated according to the Racine scale [26]. *Status Epilepticus* (SE) was defined as continuous seizures with a Racine score of 3 to 5, without returning to lower stages for at least 5 minutes. Pilocarpine-treated animals were divided into two groups (SE and No SE) depending on their behavioral response, as described below in the “Results” section. Approximately 70% of the pilocarpine treated rats showed acute behavioral features of SE (SE group) between 40–60 minutes after pilocarpine injection. Thirty percent of the animals that were injected with pilocarpine did not develop SE, showing only behavioral signs corresponding to stages 1–2 of the Racine score and were considered as a separate experimental group (No SE). All animals received 20 mg/kg diazepam 15 or 30 minutes after the onset of SE and doses were repeated as needed to terminate seizures. SE duration was limited to 30 min to reduce animal mortality, considering that it was described that 15–30 min are enough for establishing a chronic period of spontaneous seizures [25]. To study cell morphology, different time points were established: 3, 7, 15 or 21 days post-SE (DPSE). At those time points, animals were deeply anaesthetized with ketamine/xylazine (90/10 mg/kg, i.p.) and were perfused through the left ventricle as described [27]. Then, brains were cryoprotected, snap frozen and coronal 25–50 µm thick brain sections were cut using a cryostat. Free floating sections were kept in a cryoprotective solution (30% glycerol, 20% ethylene glycol in 0.05 M phosphate buffer) at −20°C until use. To study the effect of gabapentin, two treatment protocols were started 24 h after the onset of the epileptic seizures. For that purpose separate groups of animals from all experimental conditions (control, control-Li, Li-pilo No SE, Li-pilo SE) received daily gabapentin injections (400 mg/kg/day i.p) for either 4 or 14 days, or an equal volume of saline (Figure 1). Brain sections were obtained as described above.

### Immunohistochemistry

Brain sections of animals from all experimental groups were simultaneously processed in the free floating state as previously described [27]. After endogenous peroxidase activity inhibition, brain sections were permeabilized with 1% Triton X-100 in PBS, unspecific binding blocked with normal goat serum (3%) and sections incubated with the respective primary antibody. All antibodies were diluted in a solution with phosphate-buffered saline (PBS), 1% Triton X-100 and 3% normal goat serum. After 48 h incubation at 4°C, slices were rinsed and incubated with biotinylated secondary antibodies and extravidin complex. Development of peroxidase activity was carried out with 0.035% w/v 3,3′ diaminobenzidine plus 2.5% w/v nickel ammonium sulfate and 0.1% v/v H_2_O_2_ dissolved in acetate buffer 0.1 M pH 6.0. Controls for the immunohistochemistry procedure were routinely performed by omitting the primary antibody. These control sections did not develop any immunohistochemical labeling. Photographs were taken with a Zeiss Axiophot microscope (Carl Zeiss, Oberkochen, Germany) equipped with an Olympus Q-Color 5 camera. For double immunofluorescence studies, blockage of unspecific binding was performed with 10% normal goat serum. Primary antibodies were used at the following dilutions: anti-GFAP (1:1000), anti-nestin (1:800), anti-NeuN (1:1000), anti-PSANCAM (1:1000), anti-P65NLS (1:800). Fluorescent secondary antibodies conjugated with FITC or Rhodamine RRX were used at 1:800 dilution, and nuclear counterstaining was done with Hoechst 33342 (2 µg/ml). Immunofluorescence images were taken in an Olympus IX-81 microscope equipped with a DP71 camera (Olympus, Tokyo, Japan).

### Fluoro Jade B Staining

Neuronal degeneration was assessed by Fluoro Jade B staining according to the Schmued and Hopkins protocol [28]. In brief, brain sections were mounted on coated slides and immersed in a solution with 1% sodium hydroxide in 80% ethanol for 5 minutes. Slides were then immersed 2 minutes in 70% ethanol and 2 minutes in distilled water. After that, sections were exposed to a solution of 0.06% potassium permanganate for 10 min and rinsed in distilled water for 2 min. Brain sections were exposed during 20 min to a staining solution of 0.0004% Fluoro Jade B (Sigma) in 0.09% acetic acid. After rinsing for 1 min in distilled water, slides were dried at 50°C for 15 min, cleared in xylene and cover slipped.

### Dissociated Mixed Cell Culture from Postnatal Rat Hippocampus

This procedure was performed according to Lee and Parpura [29] with minor modifications. Hippocampi were obtained after brain dissection of deeply anaesthetized 3 days old Wistar rats and incubated for 1 h with papain (20 U/ml) at 37°C in 5% CO_2_. Papain was removed and tissue was washed once in DMEM. Hippocampi were mechanically dissociated with a fire-polished glass serological pipette until no visible clamps remained. Cells were plated onto poly-L-lysine-coated multiwell chambers and fed with DMEM, 1% glutamine, 1% penicillin-streptomycin and 10% fetal calf serum. Cultures were maintained at 37°C in a humidified atmosphere with 5% CO_2_; 50% of the medium was replaced by fresh medium every 3 days. This protocol was kindly provided by Dr. Vladimir Parpura (UAB, USA). Excitotoxic glutamate exposure was performed by incubating during 30 min mixed hippocampal neuroglial cultures with 500 µM glutamate. Then, medium was replaced by fresh medium including gabapentin at 5, 25, 50 or 100 µg/ml. For immunocytochemistry, cultures were washed with PBS and fixed with 4% paraformaldehyde plus 4% sucrose in PBS, pH 7.2 for 15 min at 18–25°C. Cells were then washed three times with cold PBS and permeabilized with 0.1% Triton X-100. The procedure was then followed as stated for tissue sections.

### Quantitative Studies

Morphometrical studies to analyze cell morphology and area fraction occupied by stained cells were performed using the NIH ImageJ software. Images taken with the microscope were captured with a digital camera, transformed to 8-bits gray scale, normalized, and an interactive threshold selection was carried out. Once the threshold was determined, it was kept fixed for the entire experiment. Following threshold selection, identification of the structures was performed using the software and indicating the maximal and minimal size of the expected structures (cells). Images of partial cells were excluded from the counting processes. Area fraction covered by immunostained cells was obtained with the ImageJ software. Experiments were repeated 3 times with similar results; a representative experiment is shown in the figures. A minimum of 4–5 animals per condition was used in each experiment. Statistical analysis was performed by one-way ANOVA and Student–Newman–Keuls post test. Significance was set at p<0.05 using Graph Pad Software (GraphPad Software Inc., San Diego, CA, USA). Data were presented as mean ± SEM.

## Results

### Reactive Gliosis Correlates with the Racine Scale Score Reached by the Animals

The progressive development of epileptic seizures was behaviorally monitored after lithium-pilocarpine treatment, and had its onset 40–60 min after pilocarpine injection. Seizures duration was limited to 15 or 30 min by diazepam administration. Rats treated only with lithium (control Li) did not develop behavioral changes. Animals that developed seizures were classified according to the Racine scale [26]. Early stages 1 and 2 were mostly associated with facial and oral activity. In stage 3, seizures recruited more distant structures, and forelimb clonus started. In stage 4, seizures were generalized and showed stronger clonus and rearing, and then dramatic rearing-and-falling (full stage-5 pattern), as previously described [28]. In our population, an average of 30% of the pilocarpine-injected animals showed stages 1 or 2; 50–70% of the animals showed responses ranging from stage 3 to 5, and 10% of animals died in the 3–4 days following SE. In all cases, seizures were stopped after 15 or 30 min of SE with diazepam. The animals included in the SE group were those that presented continuous seizures fluctuating from stage 3 to 5 of the Racine scale. Animals with scores of 1 or 2 were considered as a separate experimental group (No SE).

**Figure 2:**
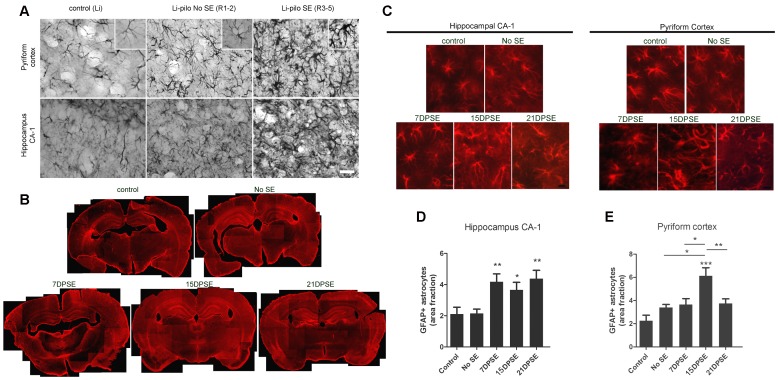
Reactive gliosis after lithium-pilocarpine induced SE. A: Astroglial hypertrophy correlates with the Racine stage reached by the animals. GFAP immunostaining showing the astroglial hypertrophy (i.e. increased soma size, larger projections and increased GFAP expression) by 7 days days after pilocarpine treatment. Control images belong to the group of lithium-saline treated rats. Bar = 70 µm. B: Low magnification images showing the reactive gliosis in the entire brain that progresses in the days following SE. Note the reduction in GFAP expression in the layer II of pyriform cortex that is recovered by 21DPSE. C: Higher magnification pictures of GFAP-positive astrocytes in the hippocampal CA-1 area and layer I of pyriform cortex. Bar = 70 µm. D: Quantitative study showing the area fraction percent occupied by GFAP-positive astrocytes in hippocampal CA-1 (D) and layers I and III of pyriform cortex (E) at different number of days post-status epilepticus (DPSE). Duration of SE: 30 min. Data on the graphs are shown as means ± SEM; significance vs. control group was represented as indicated: *p<0.05; **p<0.01; ***p<0.001 after ANOVA and Student Newman Keuls post-test.

Brain sections of the animals that presented seizures showed a profuse reactive gliosis, demonstrated by increased GFAP and nestin immunostaining. GFAP-positive astrocytes showed enlarged soma size and longer projections, together with increased GFAP expression. Figure 2A shows that reactive gliosis intensity in hippocampus and pyriform cortex correlated with the Racine stage reached by the animals. While animals at stages 1–2 showed only a minor hypertrophy, animals at stages 3–5 presented the maximal reactive gliosis by 7 days post-SE (Figure 2A). Whole brain images showed that reactive gliosis is very diffuse, affects the entire brain from 7DPSE to 21DPSE (Figure 1B) and dramatically alters astroglial cell morphology (Figure 2C). To further study the reactive gliosis pattern and to quantify the intensity, we analyzed the area occupied by GFAP immunoreactive astrocytes at different time points after the induction of epileptic seizures. An increased area fraction of GFAP-positive astrocytes was detected 7 and 15 DPSE in the hippocampal CA-1 area and pyriform cortex respectively (Figure 1C; 1D).

**Figure 3:**
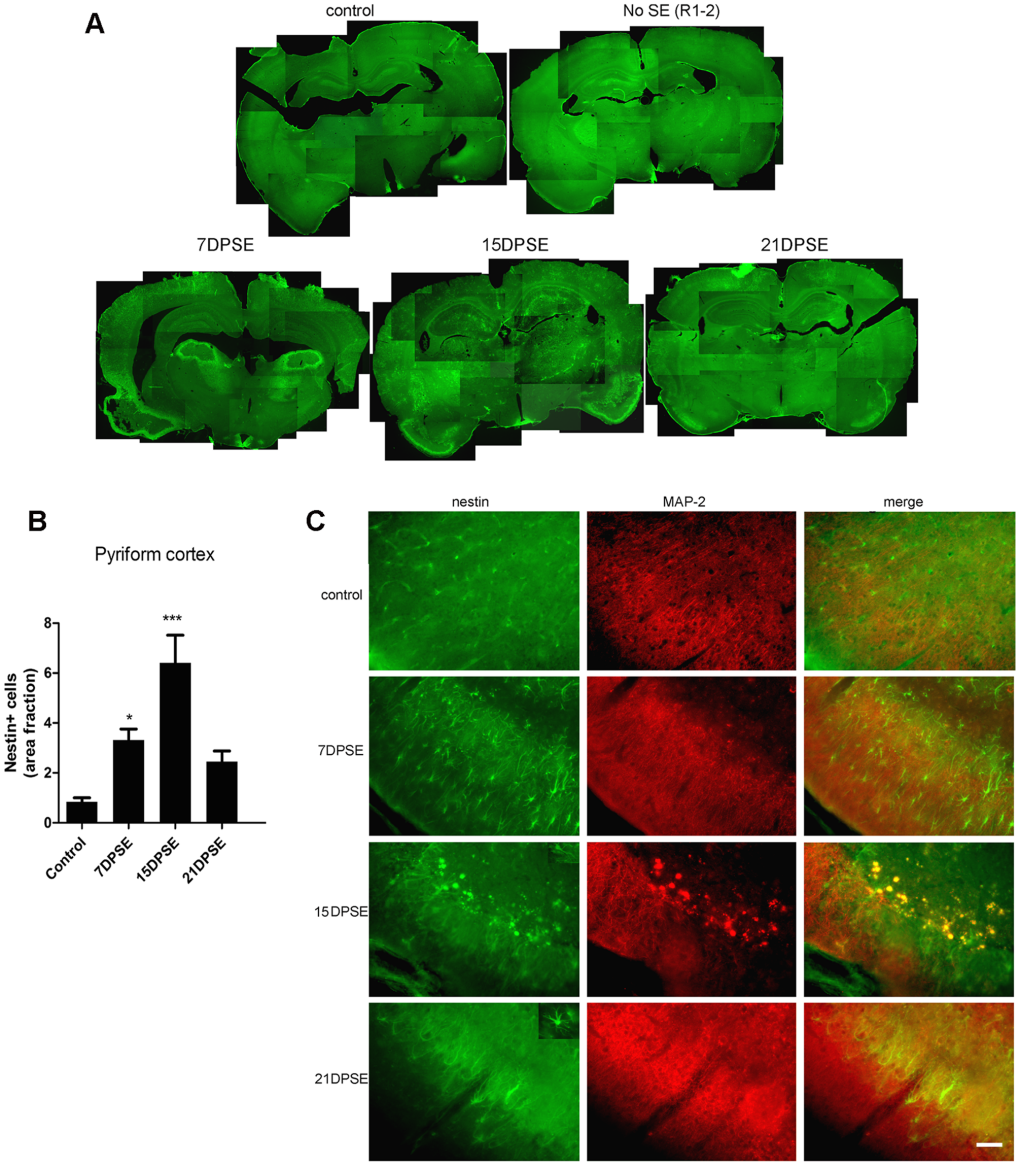
Reactive astrocytes in animals that reached SE express the immature glial marker nestin. A: Low magnification images showing the induction of nestin in the brain of animals that reached SE (Racine 3–5) but not in the animals that did not achieve SE (Racine 1–2). Note the increased nestin expression in the layer II of pyriform cortex (where GFAP was reduced in Fig. 2B). B: Quantitative study showing the area fraction percent occupied by nestin-positive cells, that peaked by 15 days post-SE (DPSE). Data on the graphs are shown as means ± SEM; *p<0.05; **p<0.01; ***p<0.001 after ANOVA and Student Newman Keuls post-test. C: Images showing the increased nestin expression in the pyriform cortex layer 2, where MAP-2 neuronal dendrites marker was lost after SE. The inset shows that nestin-positive cells have a typical glial morphology. Bar = 90 µm. The duration of the SE was 30 min.

Nestin is expressed by undifferentiated astrocytes; when found in adult animals beyond the neuro- and gliogenic niches, it is usually related to exacerbated reactive gliosis, a phenomenon that induces astrocyte de-differentiation. Nestin expression was found after pilocarpine-induced SE in the pyriform cortex of animals that reached Racine stages 3–5 (Figure 3A). The peak of maximal nestin expression was detected 15 DPSE (Figure 3B). Interestingly, this peak correlated with the reduction of MAP-2 expression in neurons of certain areas of the pyriform cortex (Figure 3C).

**Figure 4:**
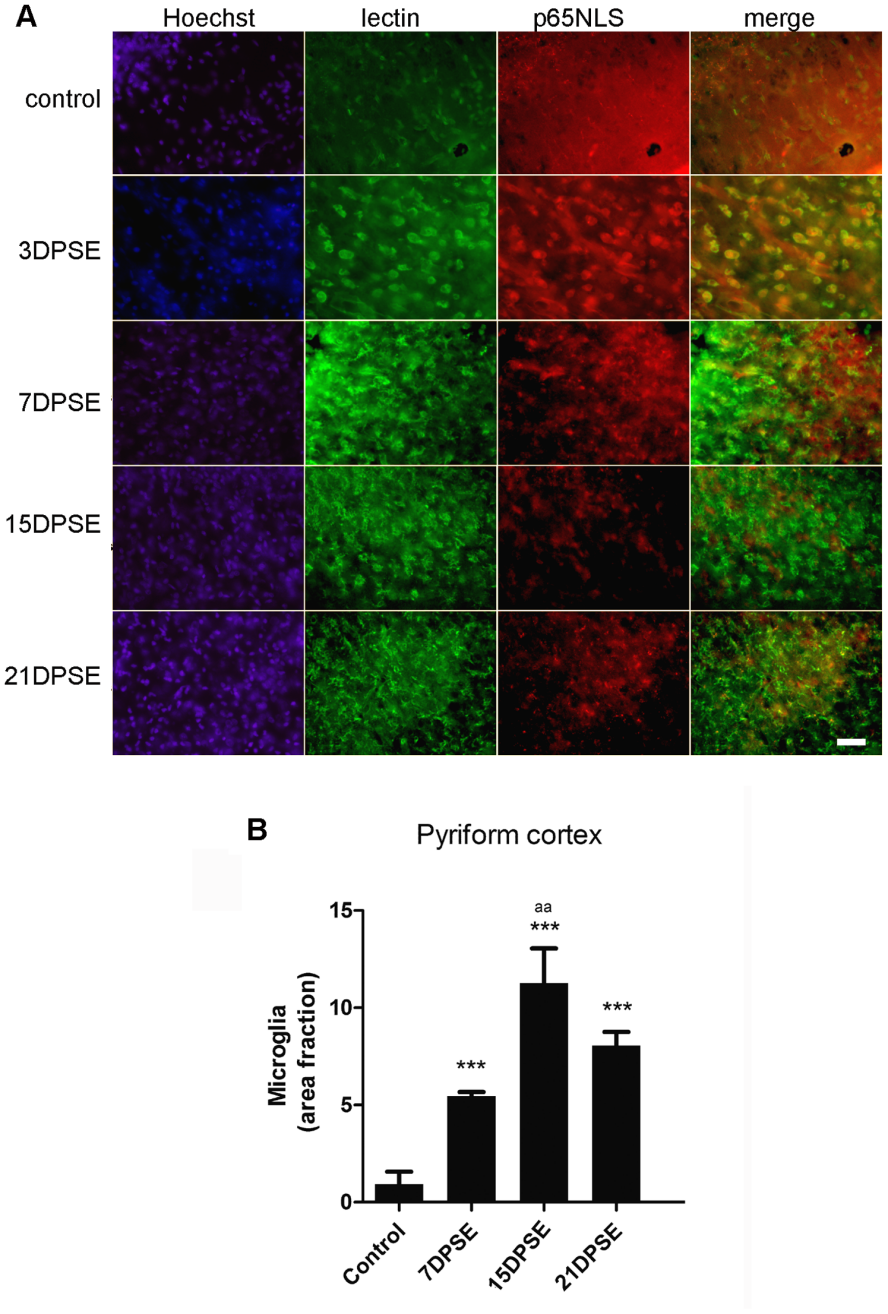
Reactive microglia is present in animals that reached SE. A: Images show the tomato-lectin positive cells in the pyriform cortex and the increased immunoreactivity for p65 NF-κB subunit exposing the nuclear localization signal (NLS). Note that reactive microglia increases during the period following SE, while p65NLS increases between 3DPSE and 7DPSE. Bar = 40 µm. B = Quantitative study showing the area fraction percent occupied by tomato-lectin positive microglia, that peaked by 15 days post-SE (DPSE). The duration of the SE was 30 min. Data on the graphs are shown as means ± SEM; significance vs. control group is represented as: *p<0.05; **p<0.01; ***p<0.001; while significance vs. 7 and 21DPSE is represented as: aa p<0.01, after ANOVA and Student Newman Keuls post-test.

Microglial cells also responded to the cellular stress induced by SE. As shown in figure 4A, reactive microglial cells identified by tomato lectin were present from 3 DPSE up to 21 DPSE. However, microglial abundance and morphology strongly changed after SE (Figure 4A, 4B). Maximal microglial reactivity peaked around 15 DPSE and was still significantly reactive 21 DPL (Figure 4B). Furthermore, NF-κB subunit p65 identified by the exposure of the p65NLS, colocalized with reactive microglia by 3DPSE (Figure 4A).

### SE-induced Neuronal Degeneration

**Figure 5:**
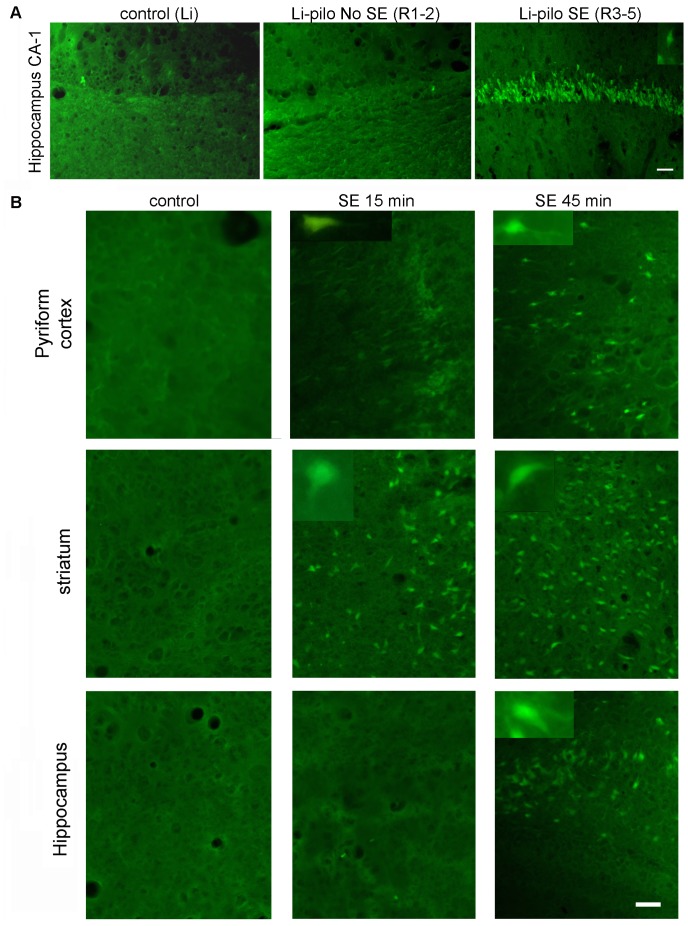
Degenerating neurons after SE. A: Degenerating neurons indentified by Fluoro-JB staining appeared in the hippocampus of animals that reached SE (Racine scale 3–5). Control images belong to the group of lithium-saline treated rats. Bar = 40 µm. B: Increasing the duration of SE from 15 min to 45 min increases the number of degenerating neurons in the pyriform cortex, striatum and hippocampus. Bar = 60 µm.

Neuronal degeneration is a well-known effect of SE induced by different experimental paradigms. In our study, the Racine stage reached by the animals correlated with the number of hippocampal neurons showing morphological signs of degeneration and positive fluoro Jade B staining by 7DPSE (Figure 5A). On the other hand, extending the duration of the SE dramatically increased the number of degenerating neurons, an effect that was still evident 14 DPSE (Figure 5B).

**Figure 6:**
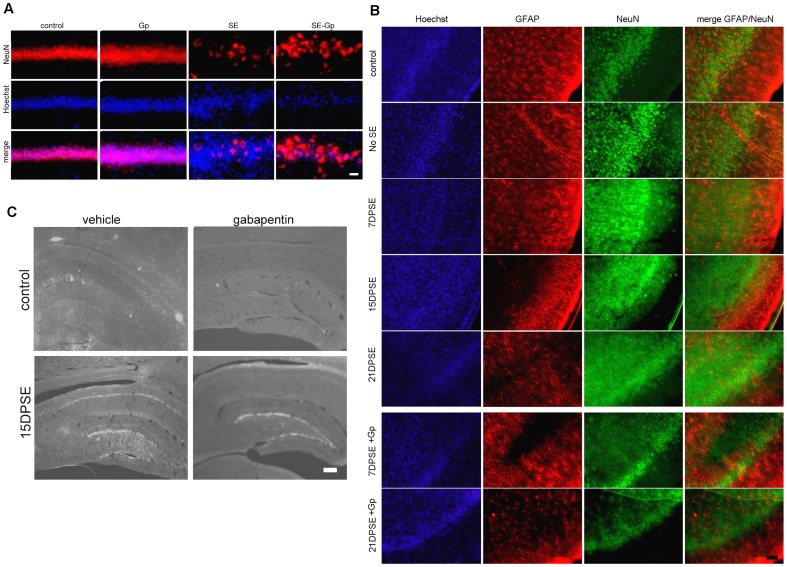
Gabapentin (Gp) treatment reduces neuronal degeneration and exacerbated plasticity induced by SE. A: Images show the loss of NeuN immunostaining and the disorganization of the CA-1 hippocampal pyramidal cell layer after 30 min of SE and the effect of 14 days treatment with daily doses of Gp. Note the partial preservation of NeuN staining in the Gp-treated animals. Control images were obtained from lithium-saline group, whereas images from Gp group were obtained from lithium-saline treated with gabapentin. Bar = 14 µm. B: The short 4-day treatment with Gp also reduced neuronal degeneration with loss of NeuN staining by 21 days post-SE (21DPSE) in the pyriform cortex. Bar = 40 µm. C: Images show the increased PSA-NCAM expression in the hippocampal CA-1 pyramidal cell layer and the dentate gyrus (DG). Only the 14-day Gp treatment was able to abolish the SE-induced PSA-NCAM expression in the CA-1 but not in the DG. Bar = 200 µm.

After 30 min of SE, neuronal degeneration could also be demonstrated by mobilization of neuronal nuclear protein NeuN from its typical nuclear localization to the cytoplasm, or even its disappearance from the neuronal soma (Figure 6A). In agreement with the previous result with FJB staining, the progressive loss of NeuN staining from 7 DPSE up to 21 DPSE (Figure 6B) evidences that neuronal degeneration is a process that progresses during the latency period after SE.

### Gabapentin Treatment Improved Neuronal Survival and Reduced Reactive Gliosis after SE

Gabapentin was originally developed as a gabaergic agonist, but subsequent studies have shown that it blocks α2δ1 neuronal receptors; reducing the synaptogenesis induced by glial-derived thrombospondin [22]. More recently it has been shown that gabapentin is also able to reduce microglial reactivity in models of hyperalgesia [24]. We therefore asked whether gabapentin would be able to reduce the neuronal and glial alterations induced by the SE. For this purpose, a group of animals that had achieved SE (Racine score >3) was daily treated with gabapentin 400 mg/kg for different periods of time (4 or 14 days after SE). Gabapentin treatment did not induce significant changes in animal survival rates to SE; however, animals treated with the drug recovered weight faster (data not shown). The 14-day gabapentin treatment partially reduced SE-induced neurodegeneration, as evidenced by loss of NeuN staining in hippocampal CA-1 pyramidal cell layer and pyriform cortex (Figure 6A). The gabapentin protective effect was even evident in the short 4-day treatment, as it partially protected pyriform cortex neurons from SE-induced degeneration (Figure 6B). The results of the 4-day treatment were still evident 21 DPSE, showing the long lasting effects of the short treatment (Figure 6B).

The neuroplasticity marker PSA-NCAM is expressed in normal brains in areas showing high rate of remodelling and neuronal plasticity. As shown in Figure 6C, PSA-NCAM is increased in hippocampal CA-1 after SE. Only the long 14-day gabapentin treatment was able to abolish the SE-induced PSA-NCAM increase in hippocampal CA-1 and CA2/3; however, this treatment was unable to affect PSA-NCAM expression in dentate gyrus (Figure 6C).

**Figure 7:**
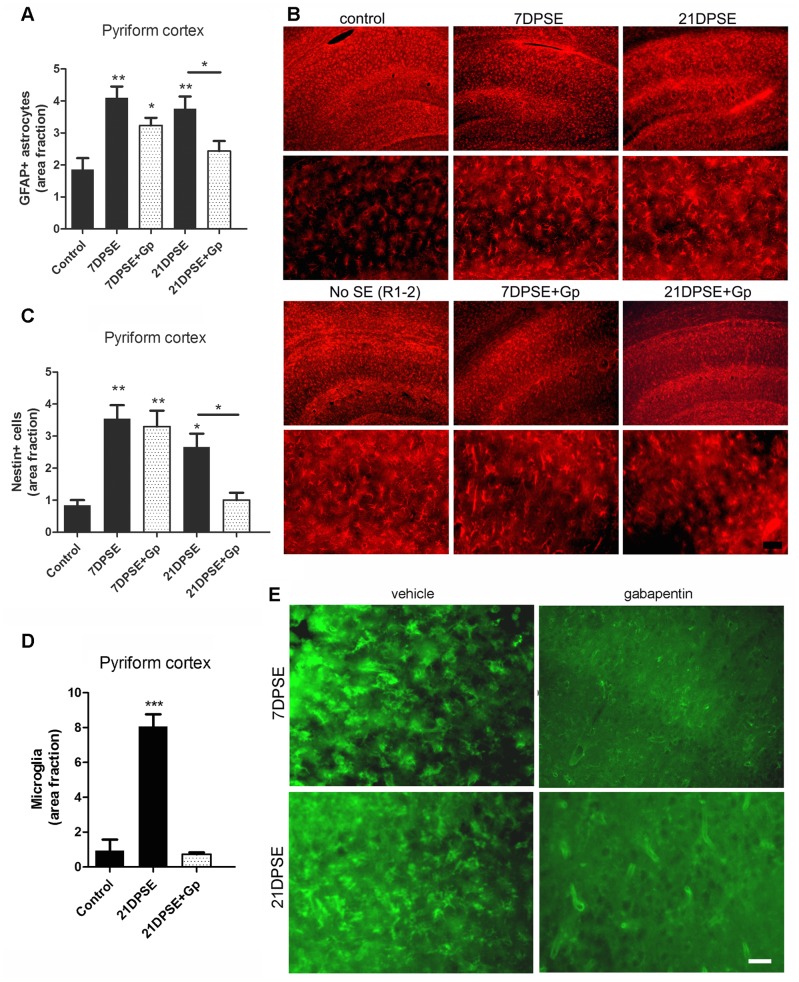
Gabapentin (Gp) treatment reduced reactive gliosis in the animals subjected to SE. A: Quantitative study showing the area fraction percent occupied by GFAP positive astrocytes in the pyriform cortex and the reduction in the area induced by the 4-day Gp treatment. B: Images show the reduction in the GFAP-positive astrocytes in the hippocampus induced by the 4-days Gp treatment. Bar = 50 µm. C: Quantitative study showing the area fraction percent occupied by nestin positive astrocytes in the pyriform cortex and the reduction in the area induced by the 4-day Gp treatment. D: Tomato-lectin positive microglia was also reduced by the 4-day Gp treatment. E: Images show the tomato-lectin positive microglial cells in the pyriform cortex, bar = 20 µm. The duration of the SE was limited to 30 min. Data on the graphs are shown as means ± SEM; significance is shown vs. control group unless indicated: *p<0.05; **p<0.01; ***p<0.001 after ANOVA and Student Newman Keuls post-test.

SE-induced reactive gliosis was also reduced by the 4-day gabapentin treatment, as shown by a reduction in the area immunostained for GFAP astroglial marker (Figure 7A), essentially due to the reduced number of astrocytes with shorter projections and smaller soma size (Figure 7B). In concordance with the reduced reactive gliosis induced by gabapentin treatment, the area fraction occupied by nestin expressing cells was reduced by 21 DPSE (Figure 7C).

Microglial cell reactivity was also reduced by the short gabapentin treatment. Figure 7D and 7E show the dramatic reduction in tomato lectin-positive microglial cells by 21 DPSE in gabapentin-treated animals.

### Gabapentin Protects Neurons and Reduces Reactive Gliosis after Glutamate-induced Excitotoxicity in vitro

**Figure 8:**
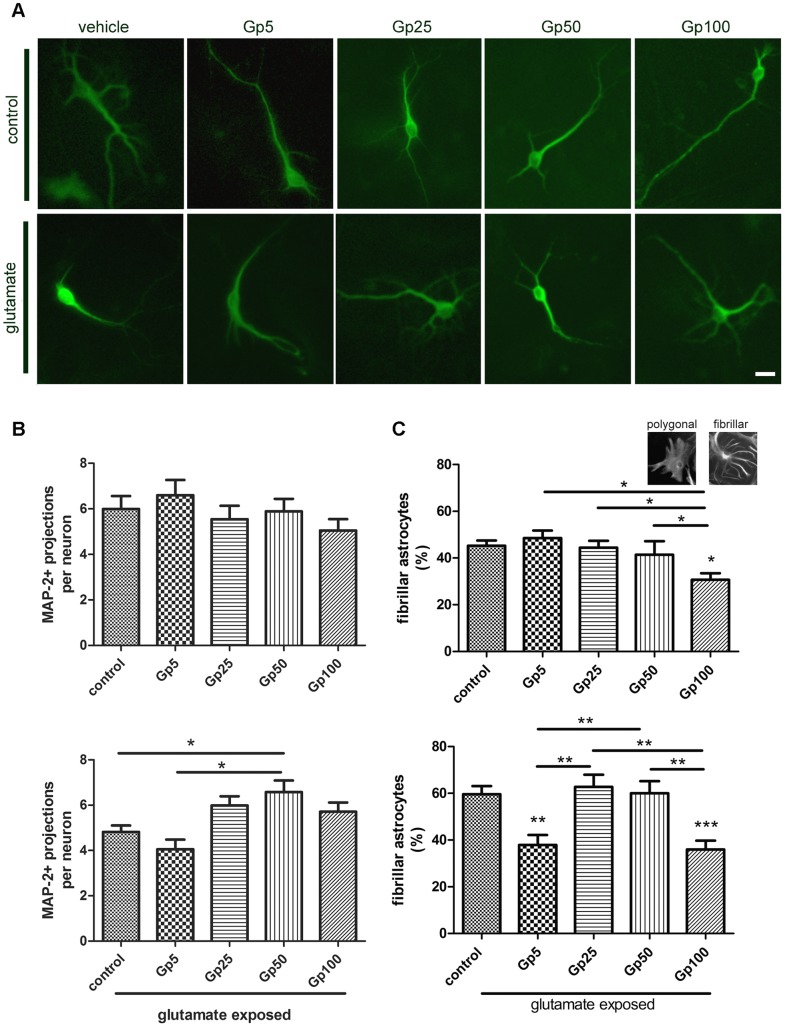
Gabapentin protects primary hippocampal neurons and reduces astroglial stellation induced by glutamate exposure. A: Representative images of neurons immunostained with MAP-2 identified in dissociated mixed cell culture from postnatal rat hippocampi exposed to glutamate and treated with gabapentin at the indicated doses for 24 h. Bar = 15 µm. B: Quantitative study showing the mean number of MAP-2 immunoreactive dendrites in hippocampal neurons in the control and glutamate-exposed group; note the prevention in the loss of projections induced by the 50 µg/ml gabapentin treatment. C: Quantitative study of the astroglial stellation in the control and glutamate exposed group; note that a low dose of gabapentin prevents astroglial stellation induced by glutamate exposure. The inset presents typical images of astrocytes in the dissociated mixed cell culture from postnatal rat hippocampi. Data on the graphs are shown as means ± SEM; significance is shown vs. control group unless indicated: *p<0.05; **p<0.01; ***p<0.001 after ANOVA and Student Newman Keuls post-test.

After evidencing the protective effect of in vivo gabapentin treatment, we wondered whether this drug could directly protect primary neurons from the excitotoxicity induced by a glutamate pulse. In order to test the hypothesis, derived from the previous results, of a glial participation in the gabapentin-induced effects, we used dissociated mixed cell culture from postnatal rat hippocampi. This culture contains most hippocampal cell types, including glial cells and neurons, and shows both the chemical and physical neuroglial interaction. To mimic the initial excitotoxic stress induced by SE, we exposed the culture to a 500 µM glutamate pulse of 30 min. Twenty four hours after the glutamate pulse; there was a reduction in the number and complexity of neuronal projections (Figure 8A, 8B). Neurons showed dendrite retraction without significant neuronal death, as assessed by nuclear morphology (data not shown), probably due to the presence of astrocytes that regulate extracellular levels of glutamate by uptake. Astroglial response to the glutamate pulse involved an increase in stellate astrocytes abundance (Figure 8C). This typical stellation phenomenon is considered as the in vitro correlation of the reactive gliosis observed in vivo [30].

The dose response gabapentin treatment showed that 50 µg/ml was the most effective to prevent the reduction in neurites number and complexity induced by the glutamate excitotoxic pulse (Figure 8B). The effect of gabapentin on control neurons not exposed to excitotoxic glutamate showed a trend to be detrimental, but it did not reached statistical significance (Figure 8B). Gabapentin exposure was also able to reduce astroglial stellation when used at the lowest dose tested (5 µg/ml) and was ineffective at 25 and 50 µg/ml (Figure 8C). The largest gabapentin dose used (100 µg/ml) presented a detrimental toxic effect both on control or glutamate-exposed dissociated mixed cell hippocampal culture (not shown).

## Discussion

Clinical data and experimental evidence indicate that epileptogenesis is a multifactorial process that comprises an initial event, usually accompanied by acute seizure activity, followed by a silent period without seizure activity. After this highly variable silent period, spontaneous seizures appear; they grow in frequency and, in some cases, become refractory to treatment (see for review [4]). A plethora of molecular events seems to occur during this period, but accumulating evidence shows that glial conversion to the proinflammatory reactive phenotype and the activation of PRR, mainly toll-like receptors, has a major role in the exacerbated neuronal irritability. Reactive glia is also probably linked to an expansion of neuronal death [31] and an altered synaptic reconnection, as it causes the release of synaptogenic molecules like TSP1–2 and hevin, which may generate spurious synapses [32]. In fact, hevin was demonstrated to be localized in excitatory synapses after pilocarpine-induced seizures [33].

In agreement with a determinant role of glial cells in the latency period following seizures, we here demonstrate that reactive gliosis initiates early after the initial SE (7 days) and reaches a peak around 15 DPSE. Astrocytes become reactive, with increased projection length and enlarged soma size both in hippocampus and pyriform cortex. Moreover, nestin, a marker of undifferentiated glial cell precursors appeared in hippocampus and pyriform cortex of animals that reached stages 3–5 of the Racine scale. The appearance of undifferentiated cells markers is usually a sign of reactive gliosis reaching the stage of severe diffuse reactive gliosis, with increased cell division and long-lasting reorganization of tissue architecture [34]. It is interesting to note that only animals that reached SE (continuous seizures with a Racine score of 3–5) presented nestin immunostaining and maximal astroglial hypertrophy detected with GFAP. These animals also showed partial loss of GFAP staining in pyriform cortex, which was replaced by nestin-positive fibrillar cells, supporting the idea of a de-differentiation of astrocytes. These de-differentiated astrocytes expressing nestin are likely to be an important source of synaptogenic molecules that may be involved in the spurious synaptogenesis proposed as the molecular basis of epileptogenesis.

Microglial cells also became reactive after SE. Reactive microglia, evaluated by the binding of tomato lectin, was present 3 DPSE, and maximal reactivity was evidenced 15 DPSE, then persisting during all the observation period (21 days). Interestingly, a significant number of microglial cells showing a reactive state also presented nuclear localization of the NF-κB p65 subunit exposing the p65NLS. This fact indicates that the NF-κB pathway, the generic output activated by DAMP/PRR signaling, is probably active in these cells.

Neuronal degeneration was known to be induced by different experimental paradigms of SE [35]; [36]; [37]; [38]; [39]; [40]. In our experimental setting, the duration of SE correlated with the detection of FJB+ degenerating cells in hippocampus, pyriform cortex and striatum. After SE, a significant loss of NeuN and MAP-2 staining was observed, which recovered during the days following SE. The loss of NeuN nuclear staining and reduction in MAP-2 positive dendrites are recognized parameters of neurodegeneration that may be to some extent reversible [27]; [41]. However, the concomitant appearance of FJB+ cells is probably showing irreversible neuronal loss. This massive neuronal degeneration is a source of DAMP, which may bind to PRR in microglia and astrocytes, the main effectors of innate immunity in the brain, and activate them.

It has been demonstrated that gabapentin efficiently blocks α2δ1 neuronal receptor, reduces TSP-induced synaptogenesis [22] and reduces epileptiform discharges in a model of post-traumatic epilepsy [23]. Gabapentin was also shown to have analgesic properties in a wide range of chronic pain models, and evidence from experimental models has shown that this effect is probably related to the gabapentin suppressive effect of microglial activation [24]; [42]. Furthermore, the activation of the PAMP/PRR pathway by the complete Freund adjuvant was able to induce the expression of the proposed gabapentin receptor α2δ1 in microglia and astroglia [24].

In our experimental model, gabapentin treatment was able to partially reduce the neuronal degeneration induced by SE. The partial inhibition of neuronal degeneration was identified as persistence of NeuN staining and reduction in FJB positive cells, especially in hippocampus. In addition, gabapentin treatment reduced the intensity of reactive gliosis, both in microglial and astroglial cells. This result is in agreement with the reported effect in experimental models of pain [24], and the partially protective action on neurons is likely to be related to the reduction in the glial conversion to the proinflammatory/neurodegenerative phenotype [43].

Interestingly, gabapentin treatment induced a significant change in the distribution of the synaptic plasticity marker PSA-NCAM. After SE, animals showed increased PSA-NCAM staining in hippocampal dentate gyrus and terminals arriving to the CA-1 pyramidal cell layer. A similar increase in PSA-NCAM immunolabeling was recently reported in pilocarpine-treated mice [44], kainic-acid treated rodents [45]; [46] and animals exposed to the amygdala kindling model of epilepsy [47]. It is believed that an increased PSA-NCAM staining reflects the exacerbated plasticity and remodeling induced in epileptic seizures that support the long-lasting changes in neural excitability. Gabapentin treatment slightly reduced the intensity of PSA-NCAM staining in DG and abolished the SE-induced increase in PSA-NCAM expression in hippocampal CA-1. It was described that gabapentin blocks the α2δ1 neuronal receptor, interfering with the binding of glial-derived TSP1–2 and impeding the formation of new excitatory synapses [22]. Gabapentin also reduces epileptiform discharges in a model of post-traumatic epilepsy [23]. In this scenario, it is reasonable to think that the gabapentin-induced changes in PSA-NCAM expression are the morphological correlate of the drug’s reported ability to block aberrant synaptogenesis that results from post-SE exacerbated neural plasticity.

In the adult brain, deafferentiation results in an initial synaptic loss, followed by a long-lasting reactive synaptogenesis where afferent axons attempt to form new synaptic connections [48]. In a model of trigeminal deafferentiation, Lo and colleages [48] have demonstrated that reactive astrocytes are involved in the induction of reactive synaptogenesis, and that blockage of astroglial purinergic signaling or TSP effects abolished reactive synaptogenesis. Since gabapentin blocks TSP1–2 but also reduces reactive gliosis, it is reasonable to speculate that gabapentin interferes with the reactive synaptogenesis induced by SE.

The protective effect of gabapentin was also observed in dissociated mixed neuroglial hippocampal cultures, where gabapentin essentially protected neurons from glutamate-induced neurite reduction, without significant effects in cultures not exposed to glutamate. However, at 100 µg/ml gabapentin had detrimental effects, probably due to its capacity to induce alterations in calcium trafficking, as reported by several groups [49]; [50]; [51]; [52]; [53]. Gabapentin also had effects on astrocytes where 5 µg/ml prevented glutamate-induced astroglial stellation. In vitro, astroglial stellation is considered a correlate of the in vivo reactive gliosis [30]. Again, 100 µg/ml gabapentin showed a detrimental effect on astrocytes. From these results obtained in dissociated mixed cell culture from postnatal rat hippocampi we determined that gabapentin has a direct effect on glutamate-exposed hippocampal neurons and astrocytes. Interestingly, the gabapentin effects seem to be evident only in glutamate-exposed cultures.

Taken together, our results indicate that gabapentin treatment during the initial phase of the silent period that follows SE was able to significantly improve different morphological parameters of neuronal damage, including mobilization of NeuN and increased PSA-NCAM expression in hippocampal fibers. In addition, gabapentin also reduced reactive gliosis in astrocytes and microglia. Based on the evidences showing that gabapentin blocks the α2δ1 receptors present both in neurons and glial cells, we propose that gabapentin reduces SE-induced brain alterations by synergistically acting on neurons, astrocytes and microglia.
